# Porphyrin Dye-Sensitized Zinc Oxide Aggregated Anodes for Use in Solar Cells

**DOI:** 10.3390/molecules21081025

**Published:** 2016-08-05

**Authors:** Yu-Kai Syu, Yogesh Tingare, Shou-Yen Lin, Chen-Yu Yeh, Jih-Jen Wu

**Affiliations:** 1 Department of Chemical Engineering, National Cheng Kung University, Tainan 701, Taiwan; hibaby403@hotmail.com (Y.-K.S.); tracymc15@gmail.com (S.-Y.L.); 2 Department of Chemistry, National Chung Hsing University, Taichung 402, Taiwan; yogeshtingare@yahoo.co.in; 3 Research Center for Sustainable Energy and Nanotechnology, National Chung Hsing University, Taichung 402, Taiwan

**Keywords:** dye-sensitized solar cells, porphyrin dyes, ZnO anode

## Abstract

Porphyrin YD2-o-C8-based dyes were employed to sensitize room-temperature (RT) chemical-assembled ZnO aggregated anodes for use in dye-sensitized solar cells (DSSCs). To reduce the acidity of the YD2-o-C8 dye solution, the proton in the carboxyl group of a porphyrin dye was replaced with tetrabuthyl ammonium (TBA^+^) in this work. The short-circuit current density (Jsc) of the YD2-o-C8-TBA-sensitized ZnO DSSCs is higher than that of the YD2-o-C8-sensitized cells, resulting in the improvement of the efficiency of the YD2-o-C8-based ZnO DSSCs. With an appropriate incorporation of chenodeoxycholic acid (CDCA) as coadsorbate, the Jsc and efficiency of the YD2-o-C8-TBA-sensitized ZnO DSSC are enhanced due to the improvement of the incident-photon-to-current efficiency (IPCE) values in the wavelength range of 400–450 nm. Moreover, a considerable increase in Jsc is achieved by the addition of a light scattering layer in the YD2-o-C8-TBA-sensitized ZnO photoanodes. Significant IPCE enhancement in the range 475–600 nm is not attainable by tuning the YD2-o-C8-TBA sensitization processes for the anodes without light scattering layers. Using the RT chemical-assembled ZnO aggregated anode with a light scattering layer, an efficiency of 3.43% was achieved in the YD2-o-C8-TBA-sensitized ZnO DSSC.

## 1. Introduction

The dye-sensitized solar cell (DSSC) is one of the most promising low-cost photovoltaic technologies. The performance of DSSCs is determined by three factors: light harvesting, electron injection, and electron collection efficiencies [1]. A sintered TiO_2_ nanoparticles (NP) film on the transparent conducting oxide (TCO) electrode—providing large surface area for dye absorption—is typically employed as the anode of DSSCs [1,2]. To achieve a high electron collection efficiency, high-temperature sintering of the TiO_2_ NP film is needed to construct a fast electron transport pathway through the NP films to TCO [1,3]. Moreover, to attain DSSCs with high efficiencies, it is crucial to develop inexpensive metal complexes or metal-free organic dyes with wide absorption profiles and appropriate highest occupied molecular orbital (HOMO)-lowest unoccupied molecular orbital (LUMO) levels to enrich the light harvesting and electron injection efficiencies [4]. Recently, a high conversion efficiency of over 14% was reported for a co-sensitized DSSC fabricated using an alkoxysilyl anchor dye (ADEKA-1) and a carboxy-anchor organic dye (LEG4) by enhancing the electron injection from the light-excited dyes to the TiO_2_ electrode [5].

Owing to its low crystallization temperature and high electron mobility, ZnO (with an energy band gap similar to that of TiO_2_) is considered as an alternative anode material for DSSCs [6,7,8,9,10]. Although ZnO possesses the advantages of rapid electron transport and high electron collection, the power conversion efficiencies of the ZnO-based DSSCs are generally lower than those of the TiO_2_-based DSSCs [1,5,6,7,8,9]. The main issue of ZnO DCCSs is that the dyes are always designed for TiO_2_ anodes, whereas there is no efficient dye available for ZnO anodes. The deterioration of ZnO and the difficulty of dye uptake during dye sensitization have been reported as the most probable reasons for the inferior performance of ZnO DSSCs [10]. This results from the poor chemical stability of ZnO in the acidic dye solution [11,12] and the presence of dye complexing agents [10]. Moreover, the influences of NPs on human health and ecological systems have been a concern in increasing the applications of NPs to commercial products [13]. Establishing effective approaches for the removal of ZnO NPs from aquatic environments has attracted considerable attention [14], which may potentially solve the issue of using ZnO NPs in large-scale DSSC application.

We have constructed a room-temperature (RT) chemical-assembled ZnO aggregated anode for use in DSSCs [9]. The aggregated ZnO anode is composed of drop-cast ZnO NPs on the indium tin oxide (ITO) substrate interconnected by the RT-grown nanostructures after a RT chemical bath deposition (CBD). By using the D149-sensitized ZnO aggregated anodes with a thickness of ~5 μm, efficiencies of 4.44% and 4.11% were monitored in the rigid and flexible ZnO DSSCs, respectively [9]. Moreover, an efficiency of 5.16% was achieved in the flexible D149-sensitized ZnO DSSC fabricated using the ZnO aggregated anode with a light-scattering ZnO particle layer prepared by the same RT chemical assembly method [9]. Although D149 exhibits high extinction coefficients, the performance of the D149-sensitized ZnO solar cells is restricted by the narrow absorption range of 400–600 nm with a tail extended to ~650 nm [6,7,8,9].

Research attention has been paid on porphyrin dyes, due to their strong absorption band covering the visible to the near-IR region, versatile modifications of their core, and facile tuning of the electronic structures [15,16,17,18,19,20,21,22,23,24,25]. An efficiency of 11.9% was reported in a zinc porphyrin (YD2-o-C8)-sensitized TiO_2_ DSSC with cobalt-based electrolyte [15]. A further increase of the DSSC efficiency to 12.3% was achieved by the co-sensitization of YD2-o-C8 with an organic dye (Y123) [15]. On the other hand, inferior performances were reported in the porphyrin-sensitized ZnO-based DSSCs [26,27]. 5-(5,15-bis(2,6-di(*n*-Hexoxy)phenyl)porphyrinato zinc(II)-2-yl)-2-carboxypenta-2,4-dienoic acid has been synthesized to sensitize ZnO nanotube electrodes, which demonstrated a DSSC efficiency of 0.5% [26]. A hematoporphyrin-sensitized ZnO nanorod photoanode has been fabricated, and an efficiency of 0.2% was acquired in the hematoporphyrin-ZnO nanohybrid DSSC [27].

In the present work, YD2-o-C8-based dyes were employed to sensitize a RT chemical-assembled ZnO aggregated anode for use in DSSCs. Due to the chemical instability of ZnO in the acidic dye solution [10,11], in the current work, the acidity of the YD2-o-C8 dye solution was reduced by replacing the proton in the carboxyl group of porphyrin dye with tetrabuthyl ammonium (TBA^+^) to form YD2-o-C8-TBA sensitizer. Co-sensitization of the RT chemical-assembled ZnO aggregated photoanode was conducted using porphyrin and indoline dyes in our previous work [28]. An efficiency of 5.6% was reported in the co-sensitized ZnO DSSC, which was optimized on the basis of panchromatic engineering. With these promising results, it is worth studying the characteristics of the YD2-o-C8-based dye-sensitized ZnO solar cells to improve the low efficiency of porphyrin-sensitized ZnO DSSCs [26,27]. Using the RT chemical-assembled ZnO aggregated anode, an efficiency of 3.43% was achieved in the YD2-o-C8-TBA-sensitized ZnO DSSC, which shows a significantly improved photovoltaic performance compared to the previously reported porphyrin-sensitized ZnO-based DSSCs [26,27].

## 2. Results and Discussion

**Figure 1:**
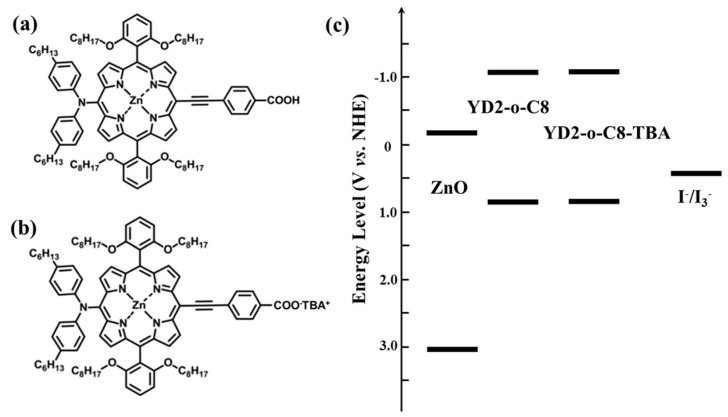
Chemical structures of (**a**) YD2-oC8 and (**b**) YD2-oC8-TBA dyes. (**c**) Energy level diagrams of ZnO, YD2-oC8, YD2-oC8-TBA, and I^−^/I_3_^−^ redox potential. (NHE: normal hydrogen electrode)

The chemical structures of YD2-o-C8 and YD2-o-C8-TBA are illustrated in Figure 1a,b, respectively. The absorption, fluorescence, and electrochemical data for the two porphyrin dyes are listed in Table 1. Figure 1c illustrates the energy level diagrams of the YD2-o-C8 and YD2-o-C8-TBA dyes, as well as their positions relative to the conduction band edge of ZnO and I^−^/I_3_^−^ redox potential. Accordingly, both porphyrin dyes possess suitable LUMO and HOMO energy levels, which ensure the energetic preferences of the photoelectron injection from the dye to the ZnO anode, as well as the dye regeneration by I^−^ in the electrolyte.

**Figure 2:**
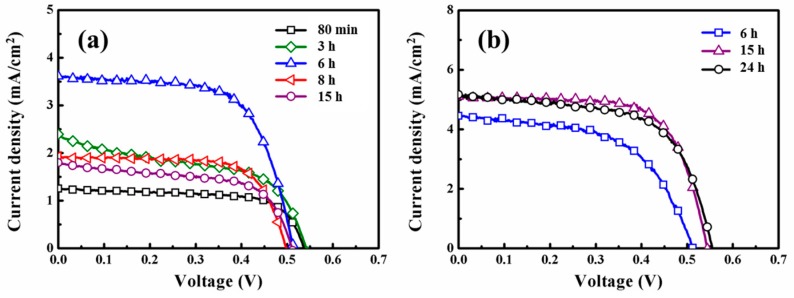
J–V curves of YD2-o-C8-sensitized ZnO dye-sensitized solar cells (DSSCs) with photoanodes fabricated with different sensitization periods in (**a**) ethanol solution of 0.4 mM YD2-o-C8; and (**b**) ethanol solution of 0.1 mM YD2-o-C8 and 0.5 mM chenodeoxycholic acid (CDCA) at room temperature (RT).

By using 5-μm-thick ZnO aggregated anodes, the influences of the period and temperature, as well as the addition of chenodeoxycholic acid (CDCA) for dye sensitization on the performance of porphyrin dye-sensitized ZnO DSSC were investigated in this study. YD2-o-C8-sensitized ZnO photoanodes were first fabricated by varying the sensitization periods in the ethanol solution of 0.4 mM YD2-o-C8 from 80 min to 15 h at RT. The DSSC performances of these YD2-o-C8-sensitized ZnO photoanodes are shown in Figure 2a and Table S1 (Supplementary Materials). As the sensitization period is elongated, the short-circuit current density (Jsc) and efficiency (η) of the DSSC first reach the peaks of 3.62 mAcm^−2^ and 1.21% in 6 h, respectively. Afterwards, decreases in both Jsc and η to plateaus are observed in the YD2-o-C8-sensitized ZnO DSSCs. Similar drops of Jsc and η were also observed in porphyrin dye-sensitized TiO_2_ DSSCs, which are attributed to porphyrin aggregation with sensitization period [22].

The addition of a coadsorbate into the dye solution has been reported to surmount the issue of dye aggregation on the TiO_2_ anode surface during sensitization [4,22]. CDCA is the most popular coadsorbate for porphyrin dyes [12,13,14,15,16,17,18,19]. In this work, the YD2-o-C8 sensitizations of the ZnO anode were further carried out in an ethanol solution of 0.1 mM YD2-o-C8 and 0.5 mM CDCA. As shown in Figure 2b and Table S1 (Supplementary Materials), with the same sensitization period of 6 h, the Jsc of YD2-o-C8-sensitized ZnO DSSC is increased from 3.62 to 4.45 mAcm^−2^ by adding CDCA in the dye solution. Moreover, the Jsc and η of YD2-o-C8-sensitized ZnO DSSC can be further improved by elongating the dye sensitization period, indicating that YD2-o-C8 aggregation is inhibited by the addition of CDCA. An efficiency of 1.89% with a Jsc of 5.11 mAcm^−2^ is acquired with a sensitization period of 15 h, as shown in Figure 2b and Table S1 (Supplementary Materials).

**Figure 3:**
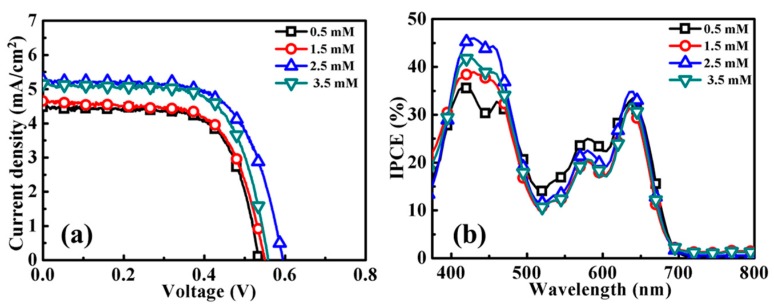
(**a**) J–V curves and (**b**) Incident-photon-to-current efficiency (IPCE) spectra of YD2-o-C8-TBA-sensitized ZnO DSSCs with photoanodes prepared using ethanol solutions of 0.1 mM YD2-o-C8-TBA with various concentrations of CDCA at RT for 15 h.

Based on the results of the YD2-o-C8-sensitized ZnO photoanodes, the non-acidic YD2-o-C8-TBA sensitized ZnO DSSCs were fabricated using 5-μm-thick ZnO aggregated anodes. Figure 3 shows the DSSC performances of the YD2-o-C8-TBA-sensitized ZnO photoanodes prepared in ethanol solutions of 0.1 mM YD2-o-C8-TBA with various concentrations of CDCA at RT for 15 h. The photovoltaic properties of these DSSC are listed in Table S2 (Supplementary Materials). With the CDCA concentration in the dye solution ranging from 0.5 to 2.5 mM (as shown in Figure 3a), the Jsc of the YD2-o-C8-TBA-sensitized ZnO DSSC is improved with increasing CDCA concentration. As 2.5 mM CDCA was added in the YD2-o-C8-TBA dye solution for the sensitization of the ZnO anode, an efficiency of 2.06% with a Jsc of 5.25 mAcm^−2^ was observed in the YD2-o-C8-TBA-sensitized ZnO DSSC. The Jsc and η are decreased when the CDCA concentration increases to 3.5 mM. The incident-photon-to-current efficiency (IPCE) spectra of the four DSSCs are shown in Figure 3b. Interestingly, IPCE values of the YD2-o-C8-TBA-sensitized ZnO DSSCs in the wavelength range of the Soret band (400–450 nm) [14] are significantly influenced by the concentration of CDCA. With an appropriate addition of CDCA, the Jsc of the YD2-o-C8-TBA-sensitized ZnO DSSC can be enhanced through the improvement of the IPCE values in the wavelength range of the Soret band, as shown in Figure 3. The results confirm the inhibition of YD2-o-C8-TBA aggregation on the ZnO anode by the incorporation of the coadsorbate CDCA.

**Figure 4:**
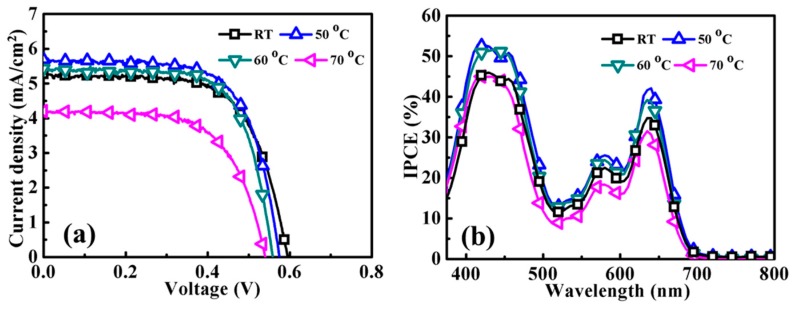
(**a**) J–V curves and (**b**) IPCE spectra of YD2-o-C8-TBA-sensitized ZnO DSSCs with photoanodes prepared using ethanol solutions of 0.1 mM YD2-o-C8-TBA and 2.5 mM CDCA for 15 h at different temperatures.

The influence of the YD2-o-C8-TBA-sensitization temperature on the ZnO DSSC performance was also studied in this work. The sensitizations of ZnO photoanodes were conducted in an ethanol solution of 0.1 mM YD2-o-C8-TBA and 2.5 mM CDCA for 15 h at different temperatures, ranging from RT to 70 °C. As shown in Figure 4a and Table S2 (Supplementary Materials), the Jsc and η of the YD2-o-C8-TBA-sensitized ZnO DSSC can be improved from 5.25 to 5.70 mAcm^−2^ and 2.06% to 2.22%, respectively, in elevating the sensitization temperature from RT to 50 °C. However, the DSSC performance of the YD2-o-C8-TBA-sensitized ZnO photoanode is degraded as the sensitization temperature is further increased. The IPCE spectra of these YD2-o-C8-TBA-sensitized ZnO DSSCs fabricated with various dye-sensitization temperatures are shown in Figure 4b. It can be seen that similar features with enhanced IPCE values are obtained in the YD2-o-C8-TBA-sensitized ZnO DSSCs as the sensitization temperature is increased from RT to 50 °C. The results show an increase in the amount of dye absorption with the sensitization temperature, resulting in the improvement of Jsc and η of the YD2-o-C8-TBA-sensitized ZnO DSSC. As shown in Figure 4b, the features of the IPCE spectra remain similar, but IPCE values reduce when the sensitization temperature is further elevated from 50 °C to 70 °C, indicating that the amount of dye absorption is optimized at a sensitization temperature of 50 °C.

**Figure 5:**
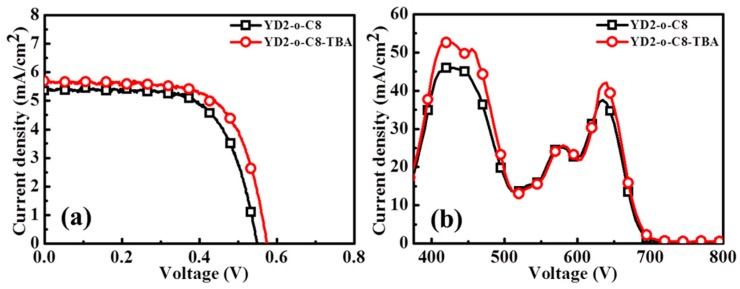
(**a**) J–V curves and (**b**) IPCE spectra of YD2-o-C8-sensitized and YD2-o-C8-TBA-sensitized ZnO DSSCs.

To study the impact of dye acidity on the performance of porphyrin-sensitized ZnO DSSCs, the YD2-o-C8-sensitized ZnO photoanodes were fabricated using the aforementioned optimized conditions for the YD2-o-C8-TBA dye sensitization—i.e., sensitization in an ethanol solution of 0.1 mM YD2-o-C8 and 2.5 mM CDCA for 15 h at 50 °C. Statistical photovoltaic parameters of the two porphyrin-sensitized ZnO DSSCs are listed in Table 2, and the J–V curves of the best cells are shown in Figure 5a. With the reduction of the YD2-o-C8 acidity, the Jsc of the YD2-o-C8-TBA-sensitized ZnO DSSCs is higher than that of the YD2-o-C8-sensitized cells, resulting in an improvement of the efficiency of the YD2-o-C8-based ZnO DSSCs. As shown in Figure 5b, the IPCE spectra of the two porphyrin-sensitized ZnO DSSCs confirm the higher IPCE values of the YD2-o-C8-TBA-sensitized cells in the wavelength ranges of 400–500 nm and 620–700 nm. It has been reported that the surface trap density of the ZnO anode is increased after sensitization in the acidic dye solution [11]. We suggest that the increased Jsc observed in the YD2-o-C8-TBA-sensitized ZnO DSSC is attributable to the increase of dye uptake and the reduction of interfacial recombination compared to the YD2-o-C8-sensitized cell.

**Figure 6:**
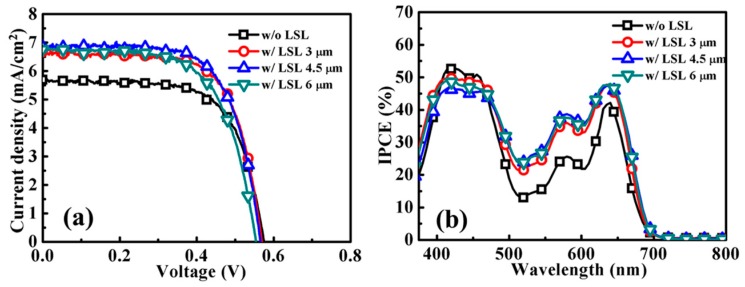
(**a**) J–V curves and (**b**) IPCE spectra of YD2-o-C8-TBA-sensitized ZnO DSSCs fabricated using 5-μm-thick ZnO anodes with light-scattering layers (LSL) of various thicknesses.

To further improve the light harvesting of the YD2-o-C8-TBA-sensitized ZnO photoanode, ZnO light scattering layers with various thicknesses were added on the 5-μm-thick ZnO anodes. The light scattering layer, consisting of ZnO particles with sizes of 200–500 nm, was drop-casted on top of the ZnO anode followed by another RT chemical bath deposition [8,9]. The J–V curves and photovoltaic properties of these YD2-o-C8-TBA-sensitized ZnO DSSCs are shown in Figure 6a and Table S3 (Supplementary Materials). Significant increases in the Jsc were achieved by the addition of a light scattering layer in the YD2-o-C8-TBA-sensitized ZnO photoanodes. The Jsc and η of the cells improved from 5.7 mAcm^−2^ and 2.22% in the absence of the light scattering layer to 6.65 mAcm^−2^ and 2.60% with a 3-μm-thick light scattering layer. The Jsc and η of the cells slightly increased at first, but then decreased with further thickening of the light scattering layer from 3 μm to 6 μm. The IPCE spectra of the cells with and without the light scattering layer are shown in Figure 6b. It shows that, compared to the cells without the light scattering layer, the IPCE values of those with the light scattering layers are considerably enhanced in the wavelengths of 475–700 nm, whereas a slight reduction of IPCE values is observed in the range of 400–475 nm. The diffuse reflectance spectra of the ZnO anodes with and without light scattering layers are shown in Figure S1 (Supplementary Materials), illustrating the improvement of the light scattering ability of the ZnO anode at wavelengths larger than 475 nm by the addition of light scattering layer. It is worth noting that significant IPCE enhancement in the 475–600 nm range is not attainable only by tuning the YD2-o-C8-TBA sensitization processes for the ZnO aggregated anodes, as shown in Figure 3b and Figure 4b. Therefore, the feature of the IPCE spectra is apparently altered with the addition of the light scattering layer on the ZnO aggregated anode.

**Figure 7:**
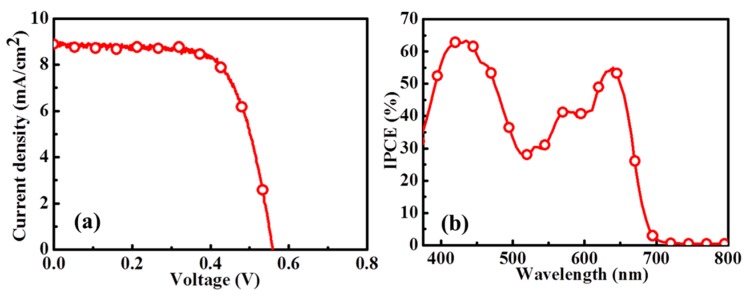
(**a**) J–V cure and (**b**) IPCE spectrum of YD2-o-C8-TBA-sensitized ZnO DSSC fabricated using 8-μm-thick ZnO anode with 3-μm-thick light-scattering layer.

Aside from the addition of a light scattering layer, another strategy for increasing the light harvesting of the YD2-o-C8-TBA-sensitized ZnO photoanode is increasing the thickness of the ZnO aggregated anode. By using the RT chemical assembly method, the maximum thickness of the uniform ZnO NP layer is ~8.3 μm. The ZnO anodes composed of an 8.3-μm-thick ZnO aggregated layer and a 3-μm-thick light scattering layer were fabricated for use in the YD2-o-C8-TBA-sensitized ZnO DSSCs. The J–V cure and IPCE spectrum of the best cell is shown in Figure 7a,b, respectively. An efficiency of 3.43% is achieved in the YD2-o-C8-TBA-sensitized ZnO DSSC. The statistical photovoltaic parameters of the YD2-o-C8-TBA-sensitized ZnO DSSCs are listed in Table 2. Compared to the performance of the cell shown in Figure 6 with the same scattering layer thickness, the Jsc increases from 6.65 mAcm^−2^ to 8.85 mAcm^−2^ by thickening the ZnO NP layer from 5 μm to 8.3 μm.

## 3. Materials and Methods

### 3.1. Preparation of ZnO Aggregated Anode

The ZnO aggregated anodes were prepared by the drop-casting of a butanolic solution of ZnO NPs with a size of 20 nm on ITO substrates, followed by a RTCBD of ZnO nanostructures to interconnect the NPs using an aqueous solution of zinc acetate and sodium hydroxide [9]. The light scattering layer with a particle size of 200–500 nm was also drop-casted on the top of the ZnO aggregated anode. The inter-necking of the particles and the connection of the light-scattering layer with the anode underneath were attained by the growth of ZnO nanostructures after another RTCBD.

### 3.2. Synthesis and Characterization of YD_2_-o-C8-TBA Salt

To a solution of YD_2_-o-C8 (100 mg, 0.065 mmol) in dry THF (3.0 mL) was added TBAOH (0.065 mL, 0.065 mmol) slowly at 0 °C. After completion of addition, the solution was stirred at room temperature for 1 h under a nitrogen atmosphere. The solvent was removed under vacuum to get the YD_2_-o-C8-TBA salt in quantitative yield. ^1^H-NMR (400 MHz, CDCl_3_) δ_H_ = 9.65 (d, *J* = 4.8 Hz, 2H), 9.13 (d, *J* = 4.8 Hz, 2H), 8.82 (d, *J* = 4.4 Hz, 2H), 8.64 (d, *J* = 4.6 Hz, 2H), 8.24 (d, *J* = 8.4 Hz, 2H), 7.94 (d, *J* = 8.4 Hz, 2H), 7.63 (t, *J* = 8 Hz, 2H), 7.19 (d, *J* = 8.4 Hz, 4H), 6.96–6.90 (m, 8H), 3.81 (t, *J* = 6.8 Hz, 8H), 3.28 (t, *J* = 8.6 Hz, 4H), 2.44 (t, *J* = 8.6 Hz, 4H), 1.67 (quin, *J* = 9 Hz, 4H), 1.51–1.41 (m, 8H), 1.25 (s, 12H), 0.99 (t, *J* = 7.2 Hz, 6H), 0.86 (t, J = 7.8 Hz, 16H), 0.81–0.72 (m, 14H), 0.62–0.41 (m, 36H). Melting point > 200 °C.

### 3.3. Fabrication and Characterization of DSSCs

Dye adsorption was conducted by immersing the ZnO aggregated anode in an ethanol solution of YD2-o-C8-based dye. The sensitized ZnO photoanode and platinized counter electrode were sandwiched together with 25-μm-thick hot-melt spacers (SX 1170-25, Solaronix SA, Aubonne, Switzerland). Electrolyte consisting of 0.05 M LiI, 0.05 M I_2_, 1.0 M 1-methyl-3-propylimidazolium iodide (PMII), and 0.5 M 4-tertbutylpyridine (TBP) in an 85:15 volume ratio of acetonitrile and valeronitrile was employed for the porphyrin dye-sensitized ZnO DSSCs.

A mask was used to create an exposed area of 0.16 cm^2^ for all cells. Photovoltaic properties of the DSSCs were measured under AM 1.5 simulated sunlight at 100 mWcm^−2^ (300 W, Model 91,160 A, Oriel, Irvine, USA). The statistical photovoltaic data of the YD2-o-C8-based ZnO DSSCs were calculated from the photovoltaic performances of 10 cells. IPCE spectra were measured using a 500 W xenon light source (Oriel) and a monochromator (Oriel Cornerstone) equipped with Si (Model 71,640, Oriel) detector.

## 4. Conclusions

In this work, porphyrin YD2-o-C8-based dyes were employed to sensitize RT chemical-assembled ZnO aggregated DSSC anodes. The proton in the carboxyl group of the porphyrin dye was replaced with TBA^+^ to reduce the acidity of the YD2-o-C8 dye solution. The Jsc, and therefore the efficiency, of the YD2-o-C8-TBA-sensitized ZnO DSSCs are higher than those of the YD2-o-C8-sensitized cells. With an appropriate addition of CDCA, the Jsc of the YD2-o-C8-TBA-sensitized ZnO DSSC can be enhanced through the improvement of the IPCE values in the wavelength range of 400–450 nm, confirming the inhibition of YD2-o-C8-TBA aggregation on the ZnO anode by the incorporation of the coadsorbate. Moreover, a significant increase of the Jsc is achieved by the addition of a light scattering layer in the YD2-o-C8-TBA-sensitized ZnO photoanodes. The IPCE values of the cell with the light scattering layers are considerably improved in the wavelengths of 475–700 nm, whereas IPCE enhancement in the 475–600 nm range is not attainable by tuning the YD2-o-C8-TBA sensitization processes for anodes without light scattering layers. Using the RT chemical-assembled ZnO aggregated anode with the light scattering layer, an efficiency of 3.43% was achieved in the YD2-o-C8-TBA-sensitized ZnO DSSC.

## Figures and Tables

**Table 1:** Absorption, fluorescence, and electrochemical data for porphyrin YD2-o-C8 and YD2-o-C8-TBA ^a^.

Porphyrin	Absorption λ_max_ (nm)	Emission λ_max_ (nm)	HOMO (V vs. NHE)	E_0-0_ (V)	LUMO (E_0-0*_) (V vs. NHE)
YD2-o-C8	448, 581, 645	663	+0.82	+1.90	−1.08
YD2-o-C8-TBA	448, 584, 639	665	+0.81	+1.91	−1.1

^a^ Absorption and emission data were measured in tetrahydrofuran at 298 K. The excitation wavelengths were 650 nm and 645 nm for YD2-o-C8-TBA and YD2-o-C8, respectively. (E_0-0_* = E_ox1_ − E_0-0_ where E_0-0_*: excited-state oxidation potential; E_0-0_: Zero-Zero excitation energy; and E_ox1_: First oxidation potential; HOMO: highest occupied molecular orbital; LUMO: lowest unoccupied molecular orbital; NHE: normal hydrogen electrode).

**Table 2:** Photovoltaic properties of YD2-o-C8-based ZnO DSSCs.

ZnO Photoanode (Dye; Thickness)		Voc (V)	Jsc (mA/cm^2^)	F.F.	η (%)
YD2-o-C8; 5 μm	Avg.	0.55 ± 0.01	5.33 ± 0.33	0.67 ± 0.03	1.94 ± 0.04
	Best	0.55	5.36	0.68	1.99
YD2-o-C8-TBA; 5 μm	Avg.	0.56 ± 0.01	5.69 ± 0.07	0.67 ± 0.01	2.14 ± 0.07
	Best	0.58	5.70	0.68	2.22
YD2-o-C8-TBA; 8 μm + light scattering layer	Avg.	0.55 ± 0.01	8.74 ± 0.19	0.68 ± 0.01	3.28 ± 0.08
	Best	0.56	8.85	0.69	3.43

## Supplementary Materials

Supplementary materials can be accessed at: http://www.mdpi.com/1420-3049/21/8/1025/s1.
