# Supplementary material for: Porphyrin Dye-Sensitized Zinc Oxide Aggregated Anodes for Use in Solar Cells
Source: Molecules. 2016 Aug 5;21(8):1025. doi: 10.3390/molecules21081025 (PMC6273904; doi:10.3390/molecules21081025)
Supplement: Supplementary file 1 [file molecules-21-01025-s001.pdf]

Supplementary Materials: Porphyrin Dye-Sensitized Zinc Oxide Aggregated Anodes for Use in Solar Cells

Yu-Kai Syu, Yogesh Tingare, Shou-Yen Lin, Chen-Yu Yeh and Jih-Jen Wu

Table S1. Photovoltaic properties of YD2-oC8-sensitized ZnO DSSCs fabricated with various dye-sensitization processes.

Sensitization Period		Voc (V)	Jsc (mA/cm²)	F.F.	η (%)
YD2-o-C8	80 min	0.54	1.25	0.68	0.45
	3 h	0.54	2.38	0.51	0.66
	6 h	0.51	3.62	0.65	1.21
	8 h	0.50	1.92	0.70	0.67
	15 h	0.51	1.80	0.60	0.55
YD2-o-C8/CDCA	6 h	0.51	4.45	0.56	1.27
	15 h	0.54	5.11	0.68	1.89
	24 h	0.56	5.17	0.62	1.78

Table S2. Photovoltaic properties of YD2-oC8-TBA-sensitized ZnO DSSCs fabricated with various dye-sensitization processes.

Sensitization		Voc (V)	Jsc (mA/cm²)	F.F.	η (%)
CDCA conc.	0.5 mM	0.54	4.47	0.70	1.68
	1.5 mM	0.55	4.66	0.66	1.70
	2.5 mM	0.60	5.25	0.66	2.06
	3.5 mM	0.56	5.15	0.68	1.97
Temperature	R.T.	0.60	5.25	0.66	2.06
	50 °C	0.58	5.70	0.68	2.22
	60 °C	0.56	5.42	0.69	2.10
	70 °C	0.54	4.18	0.64	1.45

Table S3. Photovoltaic properties of YD2-oC8-sensitized ZnO DSSCs fabricated using 5-µm-thick ZnO anodes with light-scattering layers (LSL) of various thicknesses.

Thickness of Light Scattering Layer (µm)	Voc (V)	Jsc (mA/cm²)	F.F.	η (%)
0	0.58	5.70	0.68	2.22
3.0	0.57	6.65	0.69	2.60
4.5	0.57	6.87	0.68	2.66
6.0	0.55	6.7.8	0.64	2.41

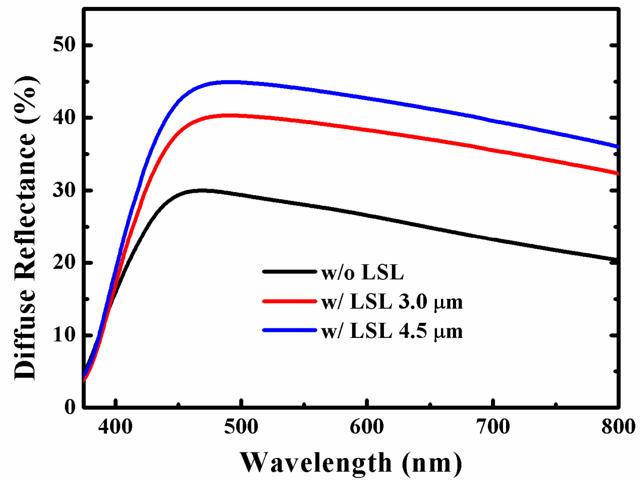

Figure S1. Diffuse reflectance spectra of the ZnO anodes with light scattering layers (LSLs) of various thicknesses.
